# Adolescents with disabilities and caregivers experience of COVID-19 in rural Nepal

**DOI:** 10.3389/fpubh.2023.1189067

**Published:** 2023-06-09

**Authors:** Joanna Morrison, Niraj Poudyal, Insha Pun, Sagar Prasai, Nir Shrestha, Dipesh Khadka, Sushmita Shrestha, Brigitte Rohwerder, Mary Wickenden

**Affiliations:** ^1^University College London Institute for Global Health, London, United Kingdom; ^2^Kathmandu University School of Arts, Kathmandu University, Lalitpur, Nepal; ^3^Diverse Patterns, Kathmandu, Nepal; ^4^Institute for Development Studies, Brighton, United Kingdom

**Keywords:** intersectional, stigma, youth—young adults, participation, South Asia, young people, disability

## Abstract

**Introduction:**

Intersecting vulnerabilities of disability, low socio-economic status, marginalization, and age indicate that adolescents with disabilities in low-and middle-income countries were uniquely affected by the COVID-19 pandemic. Yet, there has been limited research about their experience. We conducted participatory research with adolescents with disabilities in rural, hilly Nepal to explore their experience of the pandemic and inform understanding about how they can be supported in future pandemics and humanitarian emergencies.

**Methods:**

We used qualitative methods, purposively sampling adolescents with different severe impairments from two rural, hilly areas of Nepal. We collected data through semi-structured interviews with five girls and seven boys between the age of 11 and 17 years old. Interviews used inclusive, participatory, and arts-based methods to engage adolescents, support discussions and enable them to choose what they would like to discuss. We also conducted semi-structured interviews with 11 caregivers.

**Results:**

We found that adolescents with disabilities and their families experienced social exclusion and social isolation because of COVID-19 mitigation measures, and some experienced social stigma due to misconceptions about transmission of COVID-19 and perceived increased vulnerability of adolescents with disabilities to COVID-19. Adolescents who remained connected with their peers throughout lockdown had a more positive experience of the pandemic than those who were isolated from friends. They became disconnected because they moved away from those they could communicate with, or they had moved to live with relatives who lived in a remote, rural area. We found that caregivers were particularly fearful and anxious about accessing health care if the adolescent they cared for became ill. Caregivers also worried about protecting adolescents from COVID-19 if they themselves got ill, and about the likelihood that the adolescent would be neglected if the caregiver died.

**Conclusion:**

Contextually specific research with adolescents with disabilities to explore their experience of the pandemic is necessary to capture how intersecting vulnerabilities can adversely affect particular groups, such as those with disabilities. The participation of adolescents with disabilities and their caregivers in the development of stigma mitigation initiatives and strategies to meet their needs in future emergencies is necessary to enable an informed and inclusive response.

## Introduction

COVID-19 caused severe disruption to the lives of adolescents. UNICEF estimates that school closures affected more than 1.6 billion learners, with those in low- and middle-income countries having the least access to remote learning (1). There is growing concern about the longer term effects of the pandemic on adolescents’ health, well-being, literacy, income and professional opportunities (2). Adolescence is a time of psychological and social transformation. At puberty, parent–child relationships evolve as adolescents seek more independence and autonomy, and both peers and parents become reference points for adolescents as they learn to deal with more intense emotions (3). Research shows that peer influences on health and well-being are greater in adolescence than at any other time in the life course (4, 5). Peer interaction is important to develop cognitive abilities to navigate social networks and understand others’ perspectives (6). Physical distancing as part of COVID-19 control measures has meant that many adolescents have been socially isolated at this crucial time in their social development. It is likely that the effect of COVID-19 has been amplified for adolescents with disabilities, who are more likely to live in poverty without access to the internet, who are less likely to attend school, and are more likely to experience social exclusion than adolescents without disabilities (7). Our qualitative research explores and reflects on the experience of adolescents with disabilities in rural Nepal and adds to the literature in developing an inclusive understanding of the effect of the COVID-19 pandemic on adolescents with disabilities.

### Experiences from low-and middle-income countries

There are few studies on the adolescent experience of the pandemic from low-and middle-income countries, and even fewer about the experiences of adolescents with disabilities. Our recent scoping review found only 30 studies from low- and middle-income countries in the gray and academic literature about the experiences of adolescents with disabilities (8). These studies showed that lockdowns, school closures, isolation, food insecurity, economic pressures, and disruption to life during the pandemic meant that many were bored, sad, stressed, anxious, angry, and suicidal (9–14). A multi-country study examining the differences between adolescents with disabilities and their peers without disabilities found that adolescents with disabilities were more likely to lose sleep, be more distressed and engage in aggressive behaviors than adolescents without disabilities during the COVID-19 pandemic (11). While not being extensive, the literature demonstrated the importance of context-specific research to understand adolescents’ experience to inform an inclusive response in crisis situations.

Research is needed to explore the lived experience of people with disabilities in different contexts so that global strategies can be applied appropriately in local contexts. For example there are international guidelines about inclusive and disability-aware disaster risk reduction (15, 16), but these can only be implemented with an understanding of local experiences. There is now an increasing amount of literature about the inclusion of adults with disabilities in planning for disasters and emergencies but much less about the involvement and concerns of children and adolescents with disabilities (8). Their inclusion in research about their lives is in its infancy. Adolescents with disabilities are also often excluded or overlooked in research with adolescents without disabilities (17). However, methods and approaches to meaningful inclusion of adolescents with disabilities in research are now being used more widely (18, 19).

Our research was informed by the social model of disability (20) which focuses on the response to a person’s impairment in context. The social model differentiates “impairment” from “disability.” Impairment is defined as the physical or mental condition, and disability is the discrimination and prejudice experienced by people with impairments. Disability, therefore, is a result of the social and structural environment which fails to account for impairments (21). The social model emphasizes that the experience and social significance of disability and impairment is socially constructed and varies across cultures. This model was conceptualized in the “global north” and has been criticized for being reductive, and inadequately considering the physical and mental realities of impairment for persons with disabilities in low- and middle-income countries (22). However, it has been widely applied globally and is more useful to tackle structural discrimination and disadvantage than older models.

We present the findings of our research with adolescents with disabilities which explores their experience of the pandemic in rural, hilly Nepal. We analyze and reflect on this experience to develop recommendations to better support this population during future pandemics and humanitarian emergencies. These recommendations may be conceptually generalizable to other low-and middle-income countries.

### Adolescents with disabilities in Nepal

Global estimates suggest that around 10% of children (from birth to 17 years old) have moderate to severe disabilities (23). The Nepal census reports that around 2% (513,321) of the population have a disability and 36.3% of those with disabilities have a physical disability (24). Current estimates of disability prevalence in Nepal are widely considered to underestimate actual prevalence (25, 26). For example, a survey of 18,223 households in 2014/5 reported a disability prevalence of 14.5% (27).

Adolescent-specific disability research from Nepal is scarce, though some studies have described inequalities between children with and without disabilities in Nepal before the pandemic. In 2014/5, a survey found that 35% of children with disabilities aged 5–10 years were not attending school, in comparison to only 5% of adolescents without disabilities (27). Access and retention are particularly challenging for children with disabilities, and some adolescents with disabilities attend segregated schools, despite National Plans indicating the government’s commitment to mainstreaming education for disabled children (28). Research also suggests that children with disabilities are significantly less involved in social life and face high levels of stigma and persecution (25, 29).

The Government of Nepal responded to the first cases of COVID-19 by enforcing a national lockdown from March to September 2020. This restricted public movement, limited businesses, and closed educational institutions. Restrictions were enforced again from April 2021 until August 2021 as the country endured a second wave of COVID-19 which overwhelmed health services and oxygen was in short supply. Many died from the virus and because of constrained access to health care (30). The Ministry of Health and Population reported 11,900 deaths from COVID-19 from March 2020 to February 2022. Schools reopened briefly before the second lockdown, but the long closure meant that adolescents were forced to learn at home, with very little support. Online learning was inaccessible to most adolescents with disabilities (31), and teachers felt unsupported in the implementation of online learning (32). During lockdown, education was organized for children from pre-primary to grade three from marginalized groups (including children with disabilities) through a *tole sikshya* approach. *Tole Sikshya’s* were community-based learning centers that provided educational resources and trained teachers to support learning at a neighborhood level (33). This decentralized approach to learning during the pandemic complimented online methods but was not available to adolescents with and without disabilities.

## Methods

### Setting

Data were collected from one municipality in Myagdi and one municipality in Udaypur districts in September 2021. Both districts are in the hills, with Myagdi in western Nepal and Udaypur in eastern Nepal. Myagdi has the higher Human Development Index (HDI) of the two districts (0.552), which is also above the national average HDI (0.541) (34). Myagdi benefits from the tourism industry as many visitors pass through Myagdi to get to the Himalayas. The proportion of literate women in Myagdi is slightly higher than in Udaypur (64.48% as compared with 58.2%) (34). Two and a half percent of the population in Udaypur have a disability (7,781/317,532) and 5.39% of the population in Myagdi have a disability (6,122/113,641). Myagdi has the highest rate of people living with disabilities in Nepal. Of the population with disabilities, 26% (1,592/6,122) have physical disabilities in Myagdi and 31.47% (2,449/7781) have physical disabilities in Udaypur (34). In Myagdi, 2.5% (697/27088) of adolescents aged 10–19 have a disability, and in Udaypur 2% (1,195/83740) of adolescents have a disability.

### Sampling

We sought to understand the experience of COVID-19 from a diverse range of participants, and therefore we used maximum variation sampling to purposively sample girls and boys with severe to moderate disabilities who were between the ages of 10 and 19 who had different impairment types and lived in different wards of the selected two municipalities. Two female researchers with qualitative research experience (IP and SS) were recruited and further trained in qualitative and inclusive methods. One was based in Myagdi and one in Udaypur during the study. They worked with our partner organizations, Myagdi Disabled Association, and Disabled Women Association, Udaypur, to make a list of adolescents with disabilities that met our inclusion criteria to ensure diversity in age, impairment type, gender, ethnicity, and socio-economic status. In Udaypur, a list of all children receiving the disability allowance with moderate to severe impairments was obtained from the local government office. In Myagdi, a list of adolescents with moderate to severe impairments was provided by our partner organization. This list was compiled on the basis of survey data about the disability identity card they held. Partner organization representatives in both districts called these families to explore their interest in participating in the study and arrange a time to seek formal informed consent. The sample list was revised when two adolescents with disabilities were completely unresponsive to questions when visited (one in Myagdi and one in Udaypur). These potential recruits may have needed a more fine-tuned approach and more time to participate. We sampled six adolescents per municipality. This allowed us to explore diversity of experience and complete the research within budgetary and time constraints. It was important to allow sufficient time for travel to remote areas, and for researchers to be flexible to the needs of participants. We did not aim to sample to saturation. Only one adolescent had participated in another research study about COVID-19. Eight adolescents usually attended school. Two adolescents with intellectual impairments had never been to school. Participants were between 11 and 17 years old. Seven had physical impairments including three with multiple impairments, two had visual impairments, two had intellectual impairments, two had speech impairments and two had hearing impairments.

### Recruitment

To decrease COVID-19 risks to researchers, adolescents and families, informed consent processes and data collection were completed with one or two families consecutively before moving onto to the next two families. COVID-19 risk protocols were strictly followed, and participants were asked about COVID-19 symptoms before researchers visited their house. None reported symptoms. Researchers visited the household, explained the study to caregivers and adolescents with disabilities, gave them an information sheet about the study, and sought their informed consent to participate. In one family in Myagdi, a representative from the partner organization accompanied the researcher to introduce her to the participant family. At recruitment, researchers discussed the support needs and preferences of the adolescent and the nature of their impairment. We did not formally classify impairment type.

### Data collection

Data were collected from adolescents with disabilities and their caregivers over 1 month when schools were closed due to COVID-19 restrictions (Table 1). In the last week of data collection schools began to reopen. We collected data in participants’ homes, on a veranda or inside the house, except for two hearing-impaired participants (one in Udaypur, one in Myagdi). One participant had moved to the school hostel, so we interviewed him there, and the other participant traveled to his school with his caregiver at the request of the sign language interpreter with a disability. Participants were given a cash incentive and reimbursed for travel expenses when necessary. Cash was given to adolescent participants in the presence of their guardian. We asked participants if they would like a sibling to support or accompany them during the interview. In Myagdi, one sibling was present, and in Udaypur five siblings supported five adolescents. Participants chose not to have sibling support because they felt their sibling would disturb the process or participants did not want to interrupt their sibling’s studies. Often caregivers were present during the interview despite researchers requests for privacy.

**Table 1:** Research participants.

	Female	Male	Total
Myagdi			
Caregiver	6	0	6
Adolescent	2	4	6
Udaypur			
Caregiver	5	0	5
Adolescent	3	3	6
Total	16	7	23

Researchers conducted semi-structured interviews in Nepali, and two hearing-impaired participants used adult sign language interpreters. Researchers followed a topic guide and used specifically designed tools to support inclusive data collection. They used pictures, a set of different sized and gendered dolls, illustrated story sequences, an emotions chart (where adolescents could put a thumb print to indicate an emotion), a large soft dice with buttons to indicate numbers, numbered pencils, and colored pencils and paper. Tools and pictures had been developed by artisans in Kathmandu, in consultation with the research team. The topic guide design was informed by research conducted by MW and BR in Nepal with adults with disabilities (35). The topic guide had sections about the participants’ experience of the COVID-19 pandemic, and their family and friends’ experiences. The topic guide also contained four stories about adolescents with disabilities which participants could choose to discuss. Tools (pictures illustrating possible response options, dolls representing family members, story sequences, an emotions chart, dice, and numbered pencils) were used as prompts to support the discussions. Using visual materials which the participants could take time to look at and handle was a deliberately inclusive strategy. The tools also enabled the adolescents to choose what they would like to talk about. For example, with the story sequence, they could choose between a story about a boy with disabilities or a girl with disabilities. Researchers were given guidance on the potential suitability of different tools for adolescents with different impairment types. They were encouraged to use the tools creatively and flexibly according to the preferences and needs of participants. For example, one 16-year-old participant with physical disabilities said that he did not want to use the tools at the start of the interview, and the researcher used them minimally. Data were comparable across participants. Topic guides and tools were tested in two pilot interviews in Kathmandu. We piloted methods with a severely visually impaired boy in a hostel who was 10 years old, and a 15-year-old girl who had severe physical, learning and communication disabilities, who lived with her family. Adjustments were made to some questions after piloting, to simplify the questions and remove ambiguity of one question and we discussed different ways to use the tools and pictures. Interviews took between 45 and 75 min.

To triangulate data from adolescents, we conducted semi-structured interviews with caregivers using a topic guide and some picture cards. We were not able to conduct an interview with the caregiver of a hearing-impaired participant who we interviewed in the hostel. Topic guides were piloted with caregivers of participants in Kathmandu. Caregivers were all mothers except one grandmother. Interviews with caregivers were 25–40 min long and conducted after the interview with the adolescent.

Interviews were audio recorded transcribed and translated directly from Nepali into English by a translator. Translations were checked by researchers to ensure accuracy of transcription. NP checked a sample of translations to ensure accuracy of translation. Recorded data were destroyed after transcription. IP and SS wrote a description of the data collection process and reflective notes after every household visit. These were written in English and emailed to JM who provided written and verbal feedback and advice over zoom and phone for the duration of the study.

### Analysis

We used an adapted framework approach to analysis (36). Framework follows a series of analysis steps: data familiarization, identification of a thematic framework, indexing data using the framework, charting, mapping, and identification of patterns within the data. To reflect on our methods, field researchers (IP and SS) met online with JM, MW, BR the week after data collection completion for a research methods workshop. We considered the effects of the methods and tools on the data and reflected on how our sampling affected the data, and where researchers had noted variation in the data. These reflections were thematically discussed and reported under headings of sibling involvement, data collection tools, comfort of the participants, COVID-19 protocols, similarities, and differences. Robust and in-depth discussions within our team enabled us to reach agreement about interpretations of the data. After the data were transcribed and translated, five team members read two or four translated transcripts from the adolescents and the caregivers and JM read all transcripts. All team members made notes describing the main experiences and feelings in the transcripts, and we met once to discuss what we thought was interesting and important to the participants. We also discussed example categories, as several team members were inexperienced in qualitative analysis. Team members then re-read transcripts and emailed categories they had identified in the data to JM separately who collated these into a preliminary list of themes. High level themes for adolescents and caregivers were: coping mechanisms; sources of stress; positive effects of COVID-19 and lockdown; keeping safe. JM then coded two transcripts and shared her coding with team members for comment. There was broad agreement among team members on the themes, and some sub-themes were altered and merged after discussion. JM then coded all the data in Nvivo, made charts of the findings from each transcript in thematic areas to enable comparative analysis, and wrote a descriptive summary of the findings under each theme. Charting enabled us to explore triangulation in the data and compare findings by gender, place, type of participant (caregiver or adolescent with a disability). We also looked for patterns identified by researchers during the online methods workshop. Charts and the descriptive summary were sent to team members who commented on the description, referring to existing literature, experiential knowledge and to cultural norms, which helped interpretation of findings. Team analysis is an established way to enhance rigor, particularly in cross cultural or multi-disciplinary teams (37). We were unable to member check (38) our findings with participants because we did not have time or resources to re-visit each household individually, and we did not want to increase COVID-19 risks to participants by gathering them in one location. Internet access was poor in the study area and member checking could not be facilitated online. Instead, we presented the findings to our advisory committee of representatives of disability organizations in Nepal and discussed their congruence with other research and their experiential knowledge. We also discussed the recommendations from our research with the advisory committee.

The study received ethical approval from the UCL ethics committee (4199/008) and the Kathmandu University ethics committee (73/2021).

## Results

### Access to learning

Only a few school-going participants had access to online learning. Even when there was access, this was not always inclusive. One visually impaired child said: “I did not do anything as such. I have problems attending online classes. I feel like the words are roaming around. It is difficult for me to write. I cannot see the words written far. I cannot write” (Adolescent, Myagdi). Those without access to online learning tried to study at home: “I felt bored. I read and write at home (but) it was difficult. I could not ask the teachers about things I did not know” (Adolescent, Udaypur). Although most caregivers were not overly concerned about missed learning, the adolescents were. Hearing-impaired adolescents worried particularly about losing their sign-language capacity: “I am worried that my capacity will decrease. I will forget what I have been taught, which makes me feel sad” (Adolescent, Myagdi).

### Fear of COVID-19

Most caregivers and adolescents were afraid of illness and death during the pandemic: “We were frightened. Everything was closed. People died” (Adolescent, Myagdi). One 11-year-old from Myagdi drew a picture about how he felt during COVID-19: “The person is lying in a bed. There are medicines and water.” Most participants, their families and/or close relatives and neighbors had been ill. Some did not get tested for COVID-19, and instead stayed at home and treated the symptoms with home remedies, herbal medicine, or treatments given by the local medicine shop. The costs of care-seeking were a disincentive to get tested. None of the adolescents had been very ill.

Fear of illness and death was particularly stressful for the families in our study for several reasons. Some were dependent on a breadwinner and the caregiver could not work outside the home because they were primary caregivers or disabled themselves. Other caregivers feared that no one would come to their home to look after them if they got ill, and their child was not able to look after them: “We are old, and he is a person with a disability. If we get COVID-19, we will not have anyone to feed us. We will not have anyone even to give us water to drink. No one from outside will come here. There is no way that someone from outside will enter the house. They will say they would get COVID-19” (Caregiver, Myagdi).

Other caregivers feared that no one would look after their child with disabilities while they were ill, and if a caregiver died, the adolescent would require long-term support, which was very stressful: “We were afraid, and we thought we would all die. When we would die, what would those living do?” (Caregiver, Myagdi). Others were worried about how they would travel to the hospital—both financially and logistically. This affected their care-seeking decision-making: “We can take care of ourselves, but we cannot rush him instantly to the hospital…It will take about 2 h if we walk from here, and then an hour in the vehicle. It is far. Because so many people were sick, the costs of transport were expensive. That is why we did not go for a check-up… We thought we would wait and see for a few days…. we recovered by God’s grace” (Caregiver, Myagdi). None had received government support for care-seeking.

### Stigma and social exclusion

Some adolescents with disabilities and their caregivers described their experiences of disability-related stigma and discrimination, and three participant families from Myagdi had experienced additional COVID-19 related stigma. Two adolescents in these families were physically disabled, and one was visually impaired. They were of varying socioeconomic status (one very low, one low and one middle). Adolescents with disabilities were seen as more likely to get or have COVID-19, and therefore be more likely to spread COVID-19 than adolescents without disabilities: “He is not under any medication, but villagers say they may get infected with COVID-19 from contact with him. A woman told him to get away as he may transmit COVID-19 to her while bringing fodder for the cattle. They think he already has an illness and may transmit his old disease along with COVID-19 to others” (Caregiver, Myagdi).

The experience of COVID-19 and disability-related discrimination was very stressful for adolescents. One adolescent from Myagdi said: “I felt very bad. They treated me like a Corona positive (person). They did not allow me to sit on the bench and in the vehicle. They keep telling me to stay at home.” Another said: “People blame us for spreading COVID-19. They use harsh words. We feel difficult to hear these words … They mistreat me when I go to school. They say that I spread COVID-19… They try to distance themselves from me. They tell me I will spread COVID-19… I thought we would get sick, and we would all die. (Others) make signs behind my back. They call me mad. They often pelt stones at me … this got worse during COVID-19” (Adolescent, Myagdi).

Caregivers from these three families described their experience of social isolation after they or their family members became ill. When community members did not visit them to check how they were, they felt ostracized and hated: “I wished that people would come to visit us and sit beside us and talk to us. They did not need to touch anything, but at least they could speak to us from a distance…You need a friend when you are sick. Nobody helped us… We experienced hatred from our neighbors” (Caregiver, Myagdi). One family described how neighbors had convinced the ward chairman to take her family to the town to prevent COVID-19 from spreading in the village: “They called us because the neighbors asked them to send us to the District headquarters because they were afraid we would spread COVID-19. The phone call came out of the negativity rather than concern (for us)” (Caregiver, Myagdi). This discrimination has had lasting effects on their relationships within the community: “We used to talk (with our neighbors) without any hesitation before. Now it is different” (Caregiver, Myagdi).

It appeared that mask, sanitizer, and soap distribution was not targeted according to need and some felt excluded from relief. One caregiver described feeling: “left to die” (Caregiver, Myagdi). An adolescent participant said: “They did not give sanitizer to us. First, they gave two masks and soap. They provided three masks to our family. They also asked us to buy the masks. The relief was like that” (Adolescent, Myagdi). Families of adolescents with disabilities were not socially well-connected and so received little help. Some caregivers had to demand support to obtain it: “We need to speak ahead of others to get support in hard times. Others only listen if we talk about our problems, otherwise no one will listen” (Caregiver, Udaypur). The feeling of exclusion, isolation and stress was amplified when health workers refused to visit one of our study households (Caregiver, Myagdi).

For some caregivers, the lack of support and social interaction during COVID-19 was an indication of how their adolescent would be treated after their death, which worried them. One caregiver said: “The neighborhood is like this. They will clap for you when you are ok, but will not help you when you are in trouble. No one is as good as your parents. No one is worried like your parents” (Caregiver, Myagdi). Another said: “They treated us like that when we were sick for few days. How are they going to treat my daughter when I am not there?” (Caregiver, Myagdi). Some caregivers were concerned about the psychological effects of isolating on their child when they had COVID-19. Some also found it difficult to explain the need to physically distance themselves from the adolescent when they had COVID-19.

### Social isolation and vulnerability

A few of the adolescents in our study moved residence during the pandemic. The hearing-impaired children were staying in hostels and went back to their family home, where their friends and family could not use sign language: “I only had friends who could hear. There wasn’t anyone with hearing impairment…. I missed school. I felt sad” (Adolescent, Myagdi). A few others went to live with relatives in a remote village where they did not have friends. Some adolescents with disabilities felt socially isolated as they did not live near their friends, and they missed meeting friends at school. A visually impaired girl who went to a government school said: “I feel good to study and play with friends at school. When the school closed, I felt different. I was not able to play with my friends. My friends used to take me outside during break time and bring me back to class…. Other people’s houses are far away, so there is nowhere for me to go. I cannot play with my friends because the school is closed. I cannot read and write. I feel bored staying here. I dream about when I can go to school and feel free” (Adolescent, Udaypur).

Caregivers perceived their adolescent with disabilities to be more clinically vulnerable to COVID-19, more vulnerable to mistreatment from others, and more dependent on others than those without disabilities and therefore they were particularly restrictive of their movement: “(Other children) are healthy and they can go anywhere independently, but one of my children is disabled and it was really difficult to control him during COVID-19” (Caregiver, Myagdi). The caregiver of a hearing-impaired adolescent said she found it difficult to communicate her fears to her adolescent. This was made more challenging by the fact that she did not use sign language: “It was so difficult, especially to keep him inside the house during COVID-19. I would be so stressed about him going out on his own, I would be afraid about him getting beaten by other people or him beating others” (Caregiver, Myagdi). The caregiver of an intellectually disabled girl was worried about how to access help when outside of the home, but also noted that keeping her inside had led to deterioration in her mobility: “She has a problem walking after keeping her at home for a long time during the lockdown” (Caregiver, Myagdi). Several adolescents noted the relative freedom of adults in comparison to themselves and their caregivers’ stress at letting them go outside.

Two adolescents from Myagdi and one adolescent from Udaypur who lived with larger families, or moved in with extended family, had a happier experience of the pandemic than those in smaller families: “(Me and my cousins) studied and played together at home. We met our grandparents. I met my aunt and cousin… We do not usually get to meet each other (because we live in different places). But now we can stay at home and study together” (Adolescent, Myagdi).

Access to the internet also helped adolescents to cope with the pandemic, although this was not available to all participants in our study. One physically disabled older participant who did not usually attend school appeared largely unaffected by the pandemic as he remained at home watching TV and playing games on his mobile phone (Adolescent, Myagdi). A few adolescents described similar activities and online learning with friends: “(Me and my classmates) would chat with each other during the online classes when the teacher was not there. My friends used to send a direct message which only we could see but the teacher could not” (Adolescent, Myagdi). Video chat was particularly important to hearing-impaired adolescents to connect with friends: “I felt happy about being able to video chat with my friends who were living far from me” (Adolescent, Myagdi).

## Discussion

We found that adolescents with disabilities and their families experienced social exclusion and social isolation because of COVID-19 mitigation measures. Some experienced social stigma as a result of misconceptions about increased susceptibility to COVID-19 and increased risk of transmission of COVID-19 from adolescents with disabilities. We discuss these findings in the context of the literature and make recommendations to plan for a more inclusive response in future humanitarian emergency contexts.

The social model (20) of disability was a useful framework to examine the experience of adolescents with disabilities and their caregivers. This model centers the social construction of disability and emphasizes that response to an impairment affects adolescents’ lived experience. Our data show that it was largely the attitudes and behaviors of others which negatively affected how adolescents experienced the COVID-19 pandemic. We have presented disability specific experiences which build on findings from other studies and are of particular concern as they affect adolescent well-being.

### Intersectional stigma

Intersectional stigma—the convergence of multiple stigmatized identities within a person or a group—has been documented during the COVID-19 pandemic in relation to those with disabilities (39). Examining stigma using an intersectional approach allows holistic consideration about the effects of having a stigmatized identity on people’s lived experience and health outcomes (40). Stigmatization is a process of identification and labeling of a characteristic as bad or negative, which can result in a group of people or person with the characteristic being excluded from participation in society (41). Stigma can also be driven by a process of displacement of negative emotions onto other people, situations, or things, as a response to a personal or societal threat such as the COVID-19 pandemic (42). Projection of negative feelings about the threat to others can result in stigmatization of a group of people who are labeled with the stigma. The negative characteristics associated with the threat are seen to belong to them. In our study we noted that several adolescents with disabilities and their caregivers had experienced stigma in the past, and this was exacerbated by the pandemic. Adolescents in other humanitarian emergency settings have also affected by disability-related stigma. The literature shows that their access to healthcare, education, and participation in community life was affected by stigma and resultant discrimination. For example, in Ethiopia, adolescents with disabilities reported ill-treatment while trying to access subsidized food (43).

The WHO and other multilateral organizations recognized that the lack of knowledge about transmission of COVID-19, the need to blame someone and fears about death and disease may result in certain groups being targeted, including people with disabilities (44). These agencies provided guidance early in the pandemic about how to prevent and address stigma and how to ensure that families with a member with a disability accessed the same pandemic-related support as others. We have shown that clearly more focus on early implementation of this guidance was needed (44). Engaging adolescents to inform plans to address disability-related stigma and its effect should be of primary concern - before, during and after an emergency such as the COVID-19 pandemic. There is a need to conduct more research to develop a robust evidence base about what works to reduce stigma (45).

Adolescents with disabilities were often labeled as having COVID-19 and people conflated disability with illness. People with disabilities are often seen to be sick and weak when they may not be clinically vulnerable (46). The fear that adolescents could spread the virus, the fear of contagion, was an extension of the confusion that often exists between disability and illness. It is common for disability to be seen as contagious (47) when this is almost never the case. Much of the fear and stigma that occurs around disability stems from beliefs about the causes of impairment, and the COVID-19 pandemic has added confusion and conflation to this. Additionally, the evident fear of people with disabilities and the assumption that they might have behaviors which would spread the virus demonstrates a limited understanding of the adolescents’ impairments.

Research shows that intersectional stigma consistently negatively affects health outcomes (40). Our research and other research from Nepal show that for some caregivers and people with disabilities the COVID-19 mitigation measures were more stressful than for other families (35, 48, 49). Considering their prior experience of social exclusion and its’ effects, they faced heightened distress from social distancing measures, particularly when they were ill. Access to healthcare is limited for people in rural Nepal and this difficulty is exacerbated for those with disabilities who often need additional support to reach care (50, 51). Additional stigmatized identities, such as particular caste and ethnicities which interact with disability, age and place can also impede access to care (48). The additional access barriers because of COVID-19 mitigation measures exacerbated anxiety and feelings of exclusion among caregivers and adolescents. An intersectional analysis of stigma faced by adolescents with disabilities is essential to develop a complete understanding of how the pandemic was experienced, and plan for strategies to mitigate its’ harmful effects.

### Social connectedness as protective

Our research has showed that adolescents with disabilities that fared better were those who had contact with peers, friends, and extended family and those who were able to access technology. Some literature has warned about the negative mental health impact of unsupervised and overuse of the internet and social media during the pandemic, but caregivers in our study did not report concerns. This may have been because unstable internet connections and financial constraints made prolonged and unsupervised use unlikely (32, 52). Global research has indicated that social media use during the pandemic may have had a positive effect on adolescents, enabling them to remain connected to their peers while being socially distant (6, 53). Our study and others indicate that when adolescents were unable to contact friends or peers during lockdown they felt lonely and isolated (54). In Zambia and Sierra Leone, adolescents with visual, intellectual, or multiple disabilities experienced poor mental well-being which was exacerbated by isolation and reduced social support (12). Adolescents in our study and others missed the school environment which provided an important source of social contact and learning (55, 56). While online education holds potential to facilitate learning during prolonged lockdowns for those with access to the internet, this needs to be inclusive, and ensure that adequate support for teachers, students and families is provided to optimize its’ use (57–59). Research from Jordan and Ethiopia found that students with and without disabilities had family support with their distance and online education during the pandemic, but some were forced to pause their education because it was not inclusive (55). Inequalities in utilization of online learning between adolescents with and without a disability were also reported in Ethiopia where 15% of students with disabilities were using the internet, TV or radio to continue learning during the pandemic, compared to 22% of their peers without a disability (55). Improving access to technology and a stable internet connection has benefits beyond educational achievement for adolescents, and it is important to ensure the most marginalized and hard-to-reach can access resources during emergencies.

### Caring for the caregivers

Caregivers in our study worried about the adverse effects of the pandemic on the adolescents they cared for. These concerns ranged from fears of community violence because of stigma, to prevention of transmission to the adolescent if the caregiver was ill. Their emphasis on concerns about how the adolescent and their family would cope if they were to die from COVID-19 reflect heightened stress because of increased social isolation. This finding has also been reported in other research in Nepal (35). Research in other low-, middle-, and high-income country contexts has also found that the pandemic was particularly stressful for caregivers of disabled children, who had reduced opportunities for self-care with the closure of services, and lack of social support (60–63). This stress also affected their family (64). Other research from Nepal has shown that caregivers were worried about academic losses because of prolonged school closures and lack of motivation and/or access to online learning (31). Increased support for online learning to families and teachers in the future could reduce anxiety. We found that phone contact from disabled persons organizations and health workers in times of social isolation was appreciated, and it will be important to embed this in guidance for future emergencies. Providing support to caregivers during emergencies is necessary and their participation in drafting policies and plans about the best ways to do this is recommended.

## Limitations

We had a relatively small and diverse sample of adolescents, which meant we were able to explore a breadth of experience of the pandemic, but we were unable to compare responses of adolescents from different ethnicities, ages, impairment types. We also could not explore how time since disability identification affected their experience of the pandemic. We had to use an adult sign-language interpreter with hearing-impaired adolescents as were unable to find and recruit an adolescent with the required skills. This may have affected the data collection with those participants. To minimize risk to participants, senior researchers were unable to conduct field-based support and supervision. De-briefing and frequent phone contact enabled provision of remote support, but this lack of face-to-face supervision may have affected the richness of the data.

## Conclusion

The pandemic has exacerbated pre-existing inequalities, and those already marginalized by disability, age, and low socio-economic status have been disproportionately affected (39, 65, 66). Contextually specific research is necessary to capture intersectoral experiences to enable an informed response. Recommendations from our research also reach beyond the context of Nepal and may be relevant to other low-and middle-income country contexts. Our research shows that addressing the determinants of disability-related stigma and social exclusion of adolescents with disabilities in a non-pandemic context is essential to prevent the adverse effects of future health emergencies on adolescents with disabilities. Ensuring social connectedness of adolescents to their peers and to learning are important ways to prevent social isolation and resultant depression and anxiety. Engaging caregivers in planning inclusive support strategies to deal with future health emergencies is necessary. Disaster and emergency planning must be inclusive to ensure that intersecting vulnerabilities and discrimination such as those experienced by adolescents with disabilities and their caregivers in low-income settings are not exacerbated during crises.

## Data availability statement

The datasets analysed for this study are available upon reasonable request from Dr Morrison and Dr Poudyal (joanna.morrison@ucl.ac.uk and niraj.poudyal@ku.edu.np).

## Ethics statement

This study received ethical approval from the UCL Ethics Committee (4199/088) and the Kathmandu University Ethics Committee (73/2021). Written or thumb print informed consent to participate in this study was provided by the participants’ legal guardian/next of kin, and the participant themselves.

## Author contributions

JM wrote the first draft of the manuscript and led the study. SP, NS and JM provided field support to IP and SS who collected the data. MW, BR and DK developed the tools used in the study. NP, IP, SS and BR wrote a draft of the methods section. All authors participated in data analysis and interpretation of data, and all authors read and approved the final manuscript.

## Funding

This study was funded by the UK Arts and Humanities Research Council (AH/V013459/1).

## Conflict of interest

The authors declare that the research was conducted in the absence of any commercial or financial relationships that could be construed as a potential conflict of interest.

## Publisher’s note

All claims expressed in this article are solely those of the authors and do not necessarily represent those of their affiliated organizations, or those of the publisher, the editors and the reviewers. Any product that may be evaluated in this article, or claim that may be made by its manufacturer, is not guaranteed or endorsed by the publisher.
